# Population pharmacokinetics and pharmacodynamics of sitafloxacin in patients with community-acquired respiratory tract infections

**DOI:** 10.1007/s10156-013-0580-2

**Published:** 2013-03-26

**Authors:** Yusuke Tanigawara, Mitsuo Kaku, Kyoichi Totsuka, Hiroyuki Tsuge, Atsushi Saito

**Affiliations:** 1 Department of Clinical Pharmacokinetics and Pharmacodynamics, School of Medicine, Keio University, 35 Shinanomachi, Shinjuku-ku, Tokyo, 160-8582 Japan; 2 Tohoku University Graduate School of Medicine, Sendai, Japan; 3 Tokyo Women’s Medical University, Tokyo, Japan; 4 Daiichi Sankyo Co., Ltd, Tokyo, Japan; 5 Sasebo Dojin-kai Hospital, Nagasaki, Japan

**Keywords:** Population PK–PD, Sitafloxacin, Optimal dosage regimen

## Abstract

An optimal dosage regimen of sitafloxacin was considered based on a pharmacokinetics and pharmacodynamics (PK–PD) analysis in patients with community-acquired respiratory tract infections (RTI). A population pharmacokinetic analysis of sitafloxacin was conducted using clinical data of five clinical pharmacology studies and one clinical PK–PD study in patients with RTIs. The pharmacokinetic parameters in individual patients were estimated by the Bayesian method to examine any correlation between pharmacokinetics and bacteriological efficacy. Efficacy data were obtained from the clinical PK–PD study, in which 50 or 100 mg sitafloxacin was administered twice daily for 7 days. In addition, an efficacy was simulated for a hypothetical dose regimen of 100 mg once daily. The *f*AUC_0–24h_/MIC and the *fC*_max_/MIC of sitafloxacin at a dose of 50 mg twice daily were 117.5 ± 78.0 and 7.3 ± 4.7 (mean ± SD), respectively. As a result of the univariate logistic regression analysis, the larger the value of *f*AUC_0–24h_/MIC or *fC*_max_/MIC becomes, the higher the bacteriological efficacies. The eradication rates for *f*AUC_0–24h_/MIC ≥ 30 and for *fC*_max_/MIC ≥ 2 were 96.4 % and 96.3 %, respectively. The PK–PD target values of sitafloxacin for the treatment of mild to moderate RTIs were considered to be *f*AUC_0–24h_/MIC ≥ 30 and *fC*_max_/MIC ≥ 2. The PK–PD parameters at the regimen of 50 or 100 mg twice daily in patients with RTIs reached the target values. Furthermore, a 100 mg once-daily regimen was expected to show similar efficacy based on the PK–PD simulations.

## Introduction

Sitafloxacin, ((−)-7-[(7S)-7-amino-5-azaspiro[2.4]hept-5-yl]-8-chloro-6-fluoro-1-[(*1R,2S*)-2-fluorocyclopropyl]-1,4-dihydro-4-oxo-3-quinolinecarboxylic acid), is a fluoroquinolone antimicrobial agent developed by Daiichi Sankyo Co., Ltd. in Japan. Sitafloxacin has a broad-spectrum antimicrobial activity against aerobic, anaerobic, gram-positive, and gram-negative bacteria, as well as *Mycoplasma* spp. and *Chlamydia* spp. It also has a higher antimicrobial activity than other quinolones against many pathogenic organisms [1, 2].

According to several clinical pharmacology studies that have been already reported [3–6], sitafloxacin was rapidly absorbed after oral administration and has a high bioavailability (89 %). The serum concentration of sitafloxacin increased in a dose-proportional manner between 25 and 200 mg in Japanese healthy male subjects. The cumulative urinary excretion of unchanged sitafloxacin within 48 h after administration is approximately 70 % of the dose in Japanese subjects. The renal clearance of sitafloxacin was approximately 200 ml/min, which indicates both glomerular filtration and tubular secretion are involved in the urinary elimination of sitafloxacin [7].

Recently, evidence of a correlation between the pharmacokinetics (PK) and the pharmacodynamics (PD) of antimicrobial agents has been accumulating [8–10]. These studies have reported that a clinical outcome might be predicted by the parameter indicated by the correlation between a drug concentration in the plasma/serum and the minimum inhibitory concentration (MIC) of the drug against a pathogen. Indices based on PK and PD characteristics often differ according to antimicrobial class. For example, fluoroquinolones are known as concentration-dependent agents, and bacterial eradication can be obtained by fluoroquinolones when the ratio of the area under the plasma/serum concentration–time curve (AUC) to the MIC is around 25–30 for gram-positive bacteria and around 100–125 for gram-negative bacteria [8, 11–14].

We conducted the clinical PK–PD study as phase III, and the clinical efficacy and safety of sitafloxacin have been previously reported [15]. As a result of a rough PK–PD analysis in the report, both the clinical and bacteriological efficacy of sitafloxacin for the treatment of respiratory tract infections (RTIs) were shown to be more than 90 % when either of the following target values was achieved, AUC_0–24h_/MIC ≥ 100 or *C*_max_/MIC ≥ 5. In the present report, we constructed a population PK (PPK) model of sitafloxacin using the data of RTI patients in the clinical PK–PD study and of non-patients in five clinical pharmacology studies. The PK parameters in individual patients were determined using the Bayesian method. In addition, the correlation between clinical dose regimens and the bacteriological efficacy of sitafloxacin was examined using a PK–PD analysis.

All the clinical studies described in this report were conducted in compliance with the ethical principles originating in the Declaration of Helsinki, and in compliance with the Ethics Committees of each participating hospital or institute, informed consent regulations, and the ICH Good Clinical Practices Guideline. In addition, these protocols were approved by the health authority and the institutional review board or ethics committee.

## Subjects and methods

### Subjects/patients for PPK and PK–PD analysis

A summary of the clinical trials used for the PPK analysis of sitafloxacin is shown in Table 1. Serum samples for the PK evaluation were obtained from the patients with respiratory tract infections (RTIs), mild to moderate community-acquired pneumonia, acute exacerbation of chronic bronchitis, or acute bronchitis, who were enrolled in a phase III, multi-center, open-label trial [15]. These patients received 50 or 100 mg of sitafloxacin twice daily for 7 days. The PPK model was investigated in 137 patients with RTIs as well as 75 non-patients including healthy subjects, renal-impaired subjects, and elderly subjects enrolled in either of the clinical pharmacology studies [3–6]. In these clinical pharmacology studies, subjects received a single dose (25–200 mg) or multiple doses (50 or 100 mg, twice daily, or 100 mg three times daily for 7 days) of sitafloxacin. The creatinine clearance (CL_cr_) was calculated using the Cockcroft–Gault equation [17]. The age of the subjects in the analysis ranged from 20 to 91 years; their weight ranged from 33.5 to 95.0 kg; and their creatinine clearance ranged from 16 to 178 ml/min.

**Table 1:** Summary of clinical trials of sitafloxacin used in the present analysis

Study no.	Subject description	Dosage	Number of subjects/patients	Number of serum samples	Reference
DU6859-01	Healthy male subjects (phase I)	25, 50, 100, or 200 mg, single dose	24	310	[3, 4]
DU6859-01, 02 DU6859a-03	Healthy male subjects (phase I)	50 or 100 mg, twice daily, and 100 mg, three times daily, 7 days	18	535	[4, 16]
DU6859a-19	Elderly patients with chronic respiratory tract disease (clinical pharmacology)	50 or 100 mg, single dose	10	50	[16]
DU6859a-28	Healthy male elderly or nonelderly subjects (clinical pharmacology)	100 mg, single dose	11	101	[6]
DU6859a-37	Renal-impaired subjects (clinical pharmacology)	50 mg, single dose	12	148	[5]
DU6859a-44	Patients with respiratory tract infection (phase III, PK–PD study)	50 or 100 mg, twice daily, 7 days	137	354	[15]

#### PK sampling

To estimate the PK parameters, 2 to 5 blood samples (5 ml each) were obtained from each patient at the point in the specified time windows (trough, 1–3 h after dosing, and 4–8 h after dosing) in the clinical PK–PD study. In the clinical pharmacology studies, 5 to 31 blood samples were obtained from each subject.

#### Bioanalytical method

Serum samples were stored at −20 °C until analysis. The serum concentration of sitafloxacin was measured using HPLC in the clinical pharmacology studies or LC–MS/MS in the clinical PK–PD study. The lower limit of quantification was 0.010 mg/l in both methods. In the LC–MS/MS method, 0.1 ml serum sample was diluted with a mixture of H_2_O/acetic acid (98/2). The mixture was applied to an Oasis HLB extraction cartridge (30 mg/1 ml; Waters). The intraassay accuracy and precision of the HPLC method ranged between −2.7 % and 6.2 %, and below 6.4 %, respectively, and the interassay accuracy and precision ranged between −3.0 % and 7.0 %, and below 5.0 %, respectively. The intraassay accuracy and precision for the LC–MS/MS method ranged between −1.6 % and 10.4 %, and below 6.7 %, respectively, and the interassay accuracy and precision ranged between 0.8 % and 7.8 %, and below 7.7 %, respectively.

#### Bacteriological examination

The pathogenic organisms were isolated from appropriate specimens in each patient. Ninety-one strains were isolated from the patients in the clinical PK–PD study. The MICs of sitafloxacin were measured using the broth microdilution method.

#### PPK model

The PPK analysis was performed using the nonlinear mixed-effect model (NONMEM) program (version V) with a PREDPP library and NM-TRAN preprocessor. The first-order method was used for the estimation. The PK of sitafloxacin was assumed to follow the one-compartment model with first-order absorption. The basic parameters were oral clearance (CL_t_/*F*), apparent volume of distribution (*V*_d_/*F*), and absorption constant (*k*_a_), and these parameters were estimated using a model from the PREDPP library (ADVAN2 and TRANS2).

The interindividual variability in CL_t_/*F*, *V*_d_/*F*, and *k*_a_ was modeled as follows:

$$ P_{i} = \theta \times \left( {1 + \eta_{i} } \right) $$

where *P*_*i*_ is the parameter for the *i*-th subject, *θ* is the typical value of the parameter in the population, and *η* is a random interindividual effect of a mean of 0 and a variance of ω^2^. A covariance matrix between CL_t_/*F* and *V*_d_/*F* was presumed. The residual variability was modeled as follows:

$$ C_{ij} = C^{*}_{ij} + \varepsilon_{ij} $$

where *C*_*ij*_ and *C*_ij_^*^ represent the *j*-th observed and predicted concentration for the *i*-th subject, respectively, and ε is the random intraindividual effect, which is normally distributed with a mean of 0 and a variance of σ^2^.

The effect of the covariate was evaluated using a stepwise forward addition and backward elimination process. Tested covariates were as follows: CL_cr_ and disease status on CL_t_/*F*; body weight, age, gender, and disease status on *V*_d_/*F*; fasting status and age on *k*_a_; and fasting status on *F*. A change in the objective function value of 6.64 with freedom unity represented a statistically significant (*P* < 0.01) model improvement. A new model was generated from the basic model by including covariates that were significant and which had the lowest objective function values (OFV). Based on this new model, the same procedure was repeated on the rest of the covariates. When no further covariates could be included, a backward elimination was performed at the 1 % significance level.

The bootstrap resampling procedure was repeated 1,000 times and the mean values of each parameter and the 95 % confidence intervals (2.5–97.5 %) of the final model were determined.

According to the parameters of the PPK analysis determined above, the effects of changes in the covariates on the serum concentration profile of sitafloxacin were analyzed. The steady-state serum concentration–time profiles after the repeated oral administration of 50 mg sitafloxacin twice daily were plotted for individuals who were representative of specific demographics.

#### PK exposure values of sitafloxacin in patients with respiratory tract infection

The individual PK parameters, AUC_0–24h_, *C*_max_, and trough value (12 h after administration), in the steady state after repeated oral administration twice daily were estimated using the Bayesian method. The free AUC_0–24h_ (*f*AUC_0–24h_) and free *C*_max_ (*fC*_max_) of sitafloxacin were calculated by correcting AUC_0–24h_ or *C*_max_ with unbound fraction of serum protein binding (0.388).

#### PK–PD indices for efficacy of sitafloxacin in patients with respiratory tract infection

The PK–PD analysis was performed in 74 patients whose bacteriological efficacy and MIC data for sitafloxacin against the pathogen(s) were available (91 strains). Individual *f*AUC_0–24h_/MIC and *fC*_max_/MIC values were calculated using the individual PK parameters and MIC values against the pathogens. The correlations between bacteriological efficacy and the PK–PD parameters*, f*AUC_0–24h_/MIC or *fC*_max_/MIC, were examined using univariate logistic regression analysis. In this analysis, the PK–PD parameters were treated as continuous variables. Patients were grouped according to their *f*AUC_0–24h_/MIC or *fC*_max_/MIC values, and the bacterial eradication rate was calculated for each group. The achievement rates of the *f*AUC_0–24h_/MIC and *fC*_max_/MIC target values were also calculated when sitafloxacin was administered at a dose of 50 or 100 mg twice daily. Furthermore, these PK–PD parameters at a dose of 100 mg once daily were simulated using the data of the clinical PK–PD study. The MIC values of the 91 strains in this study were combined with the PK parameters in 74 patients, and the PK–PD parameters in 6,734 patients (PK in 74 patients × MIC in 91 strains), and the attainment rates of the *f*AUC_0–24h_/MIC and *fC*_max_/MIC target values were also calculated.

## Results

### PPK parameters of sitafloxacin

**Fig. 1:**
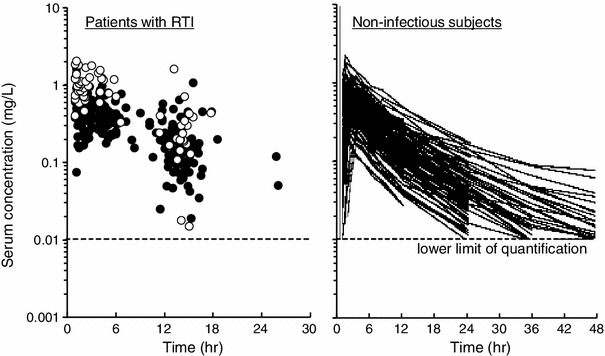
Serum concentration–time profiles of sitafloxacin in patients with respiratory tract infection (*RTI*) (*left:* *open circles* 100 mg, *n* = 28; *filled circles* 50 mg, *n* = 109; total 137 patients) and in subjects participating in clinical pharmacology studies (*right:* 25, 50, 100, or 200 mg; total 75 subjects)

A total of 1,497 serum concentration data points from 212 subjects/patients were used for the PPK analysis. The serum concentration–time profiles after the last dose are shown in Fig. 1. In the forward selection step, the CL_cr_ and disease status had a statistically significant effect on CL_t_/*F*, body weight and age on *V*_d_/*F*, and age and fasting status on *k*_a_. All the selected covariates were confirmed to be statistically significant in the backward elimination step. However, the results of a bootstrap calculation showed that the effect of age on *k*_a_ was not statistically significant, and the effect was removed from the final model. The PPK parameters for the final model, and the mean and 95 % CI of the parameters estimated using the bootstrap calculation, are shown in Table 2. The correlations between individual PK parameters estimated using the Bayesian method and covariates were examined. CL_t_/*F* appeared to be proportional to CL_cr_ with the population mean ratio of 2.58 and was 1.27 times higher in healthy volunteers than patients with RTIs. *V*_d_/*F* was correlated with body weight in a proportional manner, and the slope for the elderly patients differed from that for nonelderly patients. The *k*_a_ values after fasting were higher than those after feeding.

**Table 2:** Population pharmacokinetic parameters estimates for the final model

Parameter	Estimate (SE)	Bootstrap result
		Mean (95 % CI)
(CL_t_/*F*)/CL_cr_	2.58 (0.0810)	2.58 (2.42, 2.75)
*V*_d_/*F* (l/kg)	1.72 (0.0513)	1.73 (1.63, 1.85)
*k*_a_ (h^−1^)	1.67 (0.470)	1.86 (1.02, 3.71)
$\theta_{{{\text{status\,on\,CL}}_{\text{t}} /F}}$	1.27 (0.0881)	1.28 (1.12, 1.46)
$\theta_{{{\text{Age\,on}}\,V_{\text{d}} /F}}$	1.28 (0.103)	1.27 (1.09, 1.52)
$\theta_{{{\text{FOOD\,on}}\,k_{\text{a}} }}$	2.31 (0.410)	2.29 (1.29, 3.55)
${\omega_{{\text{CL}}_{\text{t}}}}/F^{2}$	0.0757 (0.0108)	0.0740 (0.0511, 0.0959)
${\omega_{V_{\text{d}}}}/F^{2}$	0.087 (0.0236)	0.091 (0.045, 0.150)
$\omega_{{{\text{CL}}_{\text{t}} /F, \, V_{\text{d}} /F}}$	0.0522 (0.0122)	0.0518 (0.0278, 0.0819)
${\omega}$_ka_^2^	4.57 (3.18)	6.71 (1.60, 22.65)
σ^2^	0.00923 (0.00125)	0.00901 (0.00668, 0.01150)

$$ {\text{CL}}_{\text{t}} /F({\text{l/h}}) = \left\{ \begin{array}{ll} 2.58 \times {\text{CL}}_{\text{CR}} \times 60/1,000 &({\text{patient}}) \hfill \\ 2.58 \times {\text{CL}}_{\text{CR}} \times 60/1,000 \times 1.27& ({\text{healthy volunteer}})  \end{array} \right. $$

$$ V_{\text{d}} /F({\text{l/kg}}) = \left\{ \begin{array}{ll} 1.72 \times {\text{BW}}&  ({\text{age}} < 65) \hfill \\ 1.72 \times {\text{BW}} \times 1.28 & ({\text{age}} \ge 65)\end{array} \right. $$

$$ k_{\text{a}} ({\text{h}}^{ - 1} ) = \left\{ \begin{array}{ll} 1.67& ({\text{fed}}) \\ 1.67 \times 2.31& ({\text{fasted}}) \end{array} \right. $$

*BW* body weight (kg), *CL*_*cr*_ creatinine clearance (Cockcroft–Gault; ml/min)

**Fig. 2:**
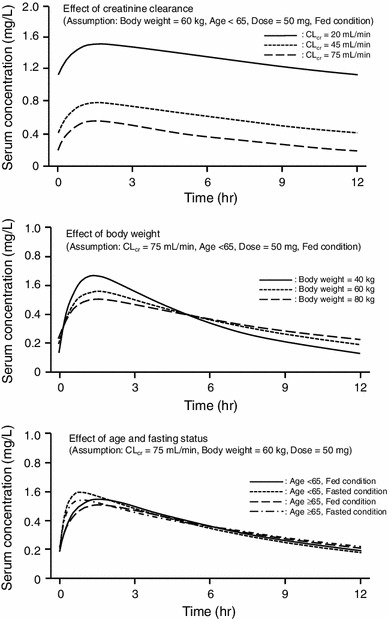
Effects of various covariates on serum concentration–time profiles of sitafloxacin. *CL*_*cr*_ creatinine clearance (Cockcroft–Gault; ml/min)

The serum concentration of sitafloxacin after the repeated oral administration of 50 mg twice daily in typical patients was estimated using the final PPK parameters. The serum concentration in patients whose CL_cr_ value was 20 or 40 ml/min was much higher than that in patients with a CL_cr_ value of 75 ml/min (Fig. 2). In contrast, a change in body weight, age, and fasting status slightly affected the serum concentration of sitafloxacin (Fig. 2). These results show that CL_cr_ is the most important factor for predicting a change in the serum concentration of sitafloxacin.

### PK exposure values of sitafloxacin in patients with respiratory tract infection

The individual PK parameters, *C*_max_, AUC_0–24h_, and trough concentration, at steady state after the repeated oral administration of sitafloxacin at a dose of 50 or 100 mg twice daily were calculated using the Bayesian method. The mean ± SD of the *C*_max_ in the 50 and 100 mg groups were 0.57 ± 0.21 and 1.17 ± 0.45 mg/l, respectively. The mean ± SD of the AUC_0–24h_ in the 50 and 100 mg groups were 9.38 ± 4.24 and 17.16 ± 6.52 mg·h/l, respectively.

### PK–PD indices for sitafloxacin in patients with respiratory tract infection

The MICs of sitafloxacin against pathogens used for the PK–PD analysis are shown in Table 3. The major isolates were *Streptococcus pneumoniae* and *Haemophilus influenzae*. The MICs of sitafloxacin against these pathogens were distributed at ≤ 0.025 mg/l and from ≤ 0.025 to 0.39 mg/l, respectively. The mean ± SD of the *fC*_max_/MIC in the 50 and 100 mg twice-daily groups were 7.3 ± 4.7 and 12.6 ± 6.4, respectively, and *f*AUC_0–24h_/MIC in the 50 and 100 mg twice-daily groups were 117.5 ± 78.0 and 198.6 ± 101.6, respectively.

**Table 3:** Pathogens isolated from patients with respiratory tract infections (91 strains)

Pathogens	Number of strains	Minimum inhibitory concentration (MIC) range of sitafloxacin (mg/l)
Gram-positive cocci		
*Staphylococcus aureus*	11	≤0.025
*Streptococcus pneumoniae*	22	≤0.025–0.39
Gram-negative bacilli		
*Moraxella catarrhalis*	12	≤0.025
*Citrobacter koseri*	1	≤0.025
*Klebsiella pneumoniae*	4	≤0.025–0.78
*Enterobacter aerogenes*	1	≤0.025
*Haemophilus influenzae*	23	≤0.025
*Haemophilus parainfluenzae*	5	≤0.025–0.39
*Pseudomonas aeruginosa*	4	0.1–0.78
*Acinetobacter calcoaceticus*	1	≤0.025
Other		
*Mycoplasma pneumoniae*	7	0.015–0.03

**Fig. 3:**
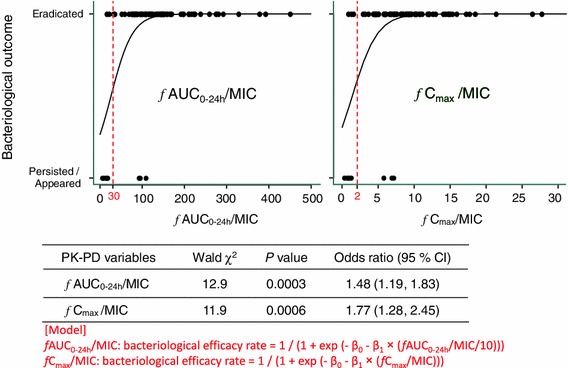
Associations between bacteriological efficacy and pharmacokinetics and pharmacodynamics (PK–PD) variables (univariate logistic regression; number of strains used for evaluation = 91)

As a result of an univariate logistic regression analysis, larger values of *f*AUC_0–24h_/MIC or *fC*_max_/MIC were shown to be correlated with a higher bacteriological efficacy of sitafloxacin. The effects of the PK–PD parameters were statistically significant (Fig. 3).

The correlation between the bacteriological efficacy of sitafloxacin and PK–PD variables in RTI patients is shown in Table 4. The eradication rate was 96.4 % when the *f*AUC_0–24h_/MIC was ≥ 30. In addition, the eradication rate was 96.3 % when the *fC*_max_/MIC was ≥ 2. In contrast, the eradication rates in the *f*AUC_0–24h_/MIC < 30 and in *fC*_max_/MIC < 2 decreased to 25.0 % and 33.3 %, respectively.

**Table 4:**
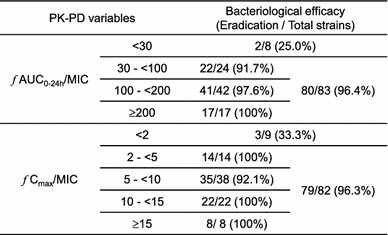
Bacteriological effect of sitafloxacin and pharmacokinetics and pharmacodynamics (PK–PD) variables in respiratory tract infections (RTIs)

The attainment of these PK–PD target values is shown in Table 5. The attainment rates of *f*AUC_0–24h_/MIC ≥ 30 and *fC*_max_/MIC ≥ 2 for the 50 mg twice-daily regimen of sitafloxacin were 90.8 % and 89.5 %, respectively. The attainment rates of *f*AUC_0–24h_/MIC ≥ 30 and *fC*_max_/MIC ≥ 2 for the 100 mg twice-daily regimen were both 93.3 %. The results of the simulation showed that the attainment rate of *f*AUC_0–24h_/MIC ≥ 30 and *fC*_max_/MIC ≥ 2 for a 100 mg once-daily regimen were also more than 90 %. Furthermore, these results were almost the same as those that compared each clinical efficacy rate (92.3 % at a dose of 50 mg; 93.1 % at a dose of 100 mg), including patients in whom the causative pathogen could not be clarified.

**Table 5:** Attainment of PK–PD target values by various dosing regimens

PK–PD target values	PK–PD study		Simulation
	50 mg twice daily	100 mg twice daily	100 mg once daily
	61 patients, 76 strains	13 patients, 15 strains	*n* = 6,734 (74 × 91)
*f*AUC_0–24h_/MIC ≥ 30	90.8 %	93.3 %	90.6 %
*fC*_max_/MIC ≥ 2	89.5 %	93.3 %	92.4 %
Clinical efficacy rate^a^	92.3 % (96/104)	93.1 % (27/29)	Not tested

^a^The clinical efficacy analysis set (*n* = 133) included patients in whom the causative pathogen could not be identified [15]

## Discussion

In the present study, we conducted a PPK analysis of sitafloxacin in patients with RTIs before an exposure–response analysis. We determined the serum concentrations of sitafloxacin in patients with RTIs enrolled in a clinical PK–PD study and analyzed these data together with data from non-patients including healthy subjects, renal-impaired subjects, and elderly subjects. The results suggested that CL_cr_, body weight, age, disease status, and fasting status influenced the PK of sitafloxacin. The CL_cr_ considerably affected the serum concentration of sitafloxacin, whereas other factors, such as body weight, age, and fasting status, had only slight effects on *C*_max_ and *T*_max_. This finding is consistent with the fact that the cumulative urinary excretion of unchanged drug after oral administration amounts to approximately 70 % in Japanese subjects [4]. Levofloxacin is primarily eliminated through the kidneys, similar to sitafloxacin, and patients with renal impairment are known to have increased serum concentration levels of these drugs [18, 19]. Therefore, a reduction in the dose or frequency of administration is recommended in renal-impaired patients.

After the repeated oral administration of sitafloxacin at a dose of 50 or 100 mg twice daily in patients with RTIs, the *C*_max_ was 0.57 and 1.17 mg/l, respectively, and the AUC_0–24h_ was 9.38 and 17.16 mg·h/l, respectively. The *C*_max_ and AUC_0–inf_ in healthy Japanese individuals treated with a 50 mg single dose of sitafloxacin were 0.51 ± 0.14 mg/l and 2.62 ± 0.53 mg·h/l, respectively [4]. The AUC_0–24h_ value that was calculated in this study was two times higher than the AUC value per dosing interval (AUC_0–tau_). However, taking this into consideration, the AUC of patients with RTIs was higher than that of healthy individuals. Renal function and creatinine clearance are known to decline with age [20]. Therefore, the elimination of drugs by renal excretion is often delayed and, consequently, an increase in the serum level is observed in elderly patients. Many elderly patients with declined renal function were enrolled in the clinical PK–PD study. Thus, the results for these subjects might have led to the higher serum concentrations of sitafloxacin compared with the results in healthy individuals.

For fluoroquinolones, *fC*_max_/MIC and *f*AUC/MIC are used to predict an antibacterial effect and the emergence of antibacterial resistance [21, 22]. We previously reported the PK–PD parameters in patients with RTIs receiving 50 or 100 mg of sitafloxacin twice daily [15]. In this previous study, the eradication rate of causative organisms increased when the *C*_max_/MIC was more than 5 and/or the AUC_0–24h_/MIC was more than 100. In the present study, we evaluated the PK–PD target value and the attainment rate of this target when 50 or 100 mg of sitafloxacin was simulated twice daily based on the results of the clinical PK–PD study. A *fC*_max_/MIC value ≥ 2 and/or a *f*AUC_0–24h_/MIC value ≥ 30 were suggested to be necessary to eradicate causative organisms in patients with RTIs. When threshold values of *C*_max_/MIC (5) and AUC_0–24h_/MIC (100) are converted to *fC*_max_/MIC and *f*AUC_0–24h_/MIC using unbound fraction of serum protein binding (0.388) of sitafloxacin, the threshold *fC*_max_/MIC and *f*AUC_0–24h_/MIC values are calculated as 2 and 39, respectively. These values are consistent with the present results for the target values of *fC*_max_/MIC (2) and *f*AUC_0–24h_/MIC (30). Actually, the 50 mg twice-daily regimen was effective for the treatment of mild to moderate community-acquired RTIs, in addition to the 100 mg twice-daily regimen. Furthermore, a 100 mg once-daily regimen of sitafloxacin was also suggested to have an efficacy similar to that of the 50 mg twice-daily regimen, based on the PK–PD simulation results.

Sitafloxacin has strong antimicrobial activity against a broad range of gram-positive and gram-negative bacteria including anaerobic bacteria, as well as against atypical pathogens. The MIC_90_ of sitafloxacin against *S. pneumoniae*, *H. influenzae*, and *Moraxella catarrhalis*, which are major pathogens in respiratory tract infections, are < 0.06, < 0.01, and < 0.01 mg/l, respectively [2]. Furthermore, sitafloxacin inhibits the activity of both the DNA gyrase and the topoisomerase IV enzymes. The inhibitory activity against these enzymes was greater than that of comparative fluoroquinolones [23]. Therefore, we consider that sitafloxacin administered orally at a dose of even 50 mg twice daily is likely to be adequately effective against major pathogens causing RTIs.

In conclusion, we conducted PPK and PK–PD analyses to estimate PK–PD parameters of sitafloxacin in patients with RTIs. The required PK–PD target values of sitafloxacin for the treatment of mild to moderate RTIs were considered to be *f*AUC_0–24h_/MIC ≥ 30 and *fC*_max_/MIC ≥ 2. The PK–PD parameters with 50 or 100 mg twice daily in major pathogens of RTIs reached these PK–PD target values for *Staphylococcus aureus*, *Streptococcus pneumoniae*, *M. catarrhalis*, *H. influenzae*, and partially for *Klebsiella pneumoniae* and *Pseudomonas aeruginosa*. Furthermore, a 100 mg once-daily regimen was expected to show similar efficacy because this regimen also reached the target values based upon the PK–PD simulations.
